# New Insight about Biocompatibility and Biodegradability of Iron Oxide Magnetic Nanoparticles: Stereological and *In Vivo* MRI Monitor

**DOI:** 10.1038/s41598-019-43650-4

**Published:** 2019-05-09

**Authors:** Hamed Nosrati, Marziyeh Salehiabar, Mohammadjavad Fridoni, Mohammad-Amin Abdollahifar, Hamidreza Kheiri Manjili, Soodabeh Davaran, Hossein Danafar

**Affiliations:** 1 Zanjan Pharmaceutical Nanotechnology Research Center, Zanjan University of Medical Sciences, Zanjan, Iran; 2 Drug Applied Research Center, Tabriz University of Medical Sciences, P.O. Box: 51656-65811, Tabriz, Iran; 3 Department of Anatomy, Medical School, Zanjan University of Medical Sciences, Zanjan, Iran; 4 Department of Anatomical Sciences and Biology, Medical School, Shahid Beheshti University of Medical Sciences, Tehran, Iran; 5 Zanjan Pharmaceutical Biotechnology Research Center, Zanjan University of Medical Sciences, Zanjan, Iran

**Keywords:** Nanoparticles, Magnetic properties and materials

## Abstract

Iron oxide magnetic nanoparticles (IONPs) have attracted enormous attention because of their extensive medicinal and industrial applicability. PEGylated L-arginine modified iron oxide magnetic nanoparticles (PEG-Arg@IONPs) were synthesized and functioned in the present research as MRI contrast agents considered *in vivo* BALB/c model. The Synthesized PEG-Arg@IONPs were tracked in certain time intervals by MRI. The intensity of MR imaging of kidneys increased after administration of PEG-Arg@IONPs, which could confirm the emission of these nanoparticles by kidneys shortly after administration. Although PEG-Arg@IONPs were uptake by liver within 2 hours after injection, whereas, the signal change intensity of spleen, heart and kidneys confirmed that PEG-Arg@IONPs existed in other organs. The results illustrated that IONPs coated with PEGylated natural amino acid thin layers had a long circulation time and could be served as T_2_ contrast agents for diagnosis purpose. Notably, to the best of our knowledge, it was the first time the biocompatibility and biodegradability of IONPs was studied and evaluated by stereological and MRI technique.

## Introduction

Iron oxide magnetic nanoparticles (IONPs) have attracted enormous attention due to their extensive application in various areas of science. IONPs are very applicable in medicine. The first MRI process was performed by Prof. Paul C. Lauterbur in 1973^1^. In 1977, Prof. Lauterbur’s method was revised by Prof. Peter Mansfield (known as line-scan technique). Prof. Peter Mansfield used this technique for the first time to image a human finger. In 1989, the U.S. Food and Drug Administration (FDA) approved the use of MRI in clinics. Later, Prof. Mansfield and Prof. Lauterbur were awarded with 2003 Nobel Prize in Physiology or Medicine for their discoveries concerning magnetic resonance imaging, which was utilized in clinics for detecting many unknown diseases^2^. There were two kinds of MRI contrast agents, i.e. positive and negative (known as T_1_ and T_2_-weighted, respectively), resulting in brighter and darker image in T_1_ and T_2_ weighted MRI, respectively^3^. In 1988, the first marketed MRI contrast agent, Magnevist^®^ (Gd-DTPA), was presented. At this time, positive contrast agents, including Gadovist^®^ (Gd-DO3A-Butriol), Eovist^®^ (Gd-EOB-DTPA), ProHance^®^ (Gd-DO3A-HP), Multihance^®^ (Gd-BOPTA), OptiMARK^®^ (Gd-DTPA-BMEA), Dotarem^®^ (Gd-DTOA), and Omniscan^®^ (Gd-DTPA-BMA) were commercialized and called gadolinium chelates^3^. However, new studies have shown, these types of chelates can increase the chance of nephrotoxicity *in vivo*^4–6^. Therefore, an universal warning FDA was circulated regarding the toxicity of gadolinium based compounds^7^ and because of the biocompatibility of IONPs, these types of contrast agents have been increasingly applied in MRI.

Recently, IONPs have been widely considered in medical and pharmaceutical fields as MRI contrast agents^8–12^. Some IONPs marketed as commercial products are accessible as MRI contrast agents. The reticuloendothelial system destroys the administrated IONPs shortly after injection selectively. Hence, the IONPs circulation time is generally short.

Therefore, prolongation of IONPs circulation times is an interesting technical issue. Researchers have used hydrophilic polymers such as poly (ethylene glycol) (PEG) to coat IONPs and prolong the circulation time^13–19^.

Numerous studies have been reported about the behavior of IONPs *in vivo*^20,21^. However, the overall relationship between IONPs surface and their bio-distribution has remained unclear. Thus, IONPs surface engineering was needed for popular biomedical uses^22–24^. Finally, although the literature review can provide a considerable amount of information about the effect of IONPs on the living systems, but, it is still not adequate to reach overall conclusions. Consequently, *in vivo* evaluation of toxicity factors by new techniques may bring a good information to the ongoing discussion about bio-safety of nanomaterials.

We used biocompatible and nontoxic small natural molecules, i.e. Arginine amino acid, as a IONPs capping agent (noted as Arg@IONPs). In the present work, we conjugated polyethylene glycol (PEG) on the surface of Arg@IONPs (PEG-Arg@IONPs) in order to enhance the colloidal stability and prolong the blood circulation time. Characteristics of the particles were analyzed by different techniques. The biocompatibility and stereological study had been performed for more than 15 days after nanoparticles administration, using hystomorphometric analysis. In addition, overall biodegradability and blood clearance of PEG-Arg@IONPs were studied by MRI techniques in an animal model. The MRI signal intensity have been recorded for 24 h after injection. Histology results obtained from liver, kidney, heart, and spleen specimens were also affected by the pathological changes induced by IONPs. Significantly, to the best of our knowledge, this is the first biocompatibility and biodegradability study of IONPs using stereological and MRI techniques, respectively.

## Materials and Methods

All methods used in this study were conducted in accordance with relevant guidelines and regulations of the Ethics Committee of Zanjan University of Medical Sciences under IR.ZUMS.REC.1396.307 ethical code.

### Materials

FeCl_3_.6H_2_O, 3-(4,5-dimethylthiazol-2-yl)-2, 5-diphenyl tetrazolium bromide (MTT), FeCl2.4H_2_O, Polyethylene glycol monomethyl ether (mPEG, MW = 4 kDa), ammonium hydroxide, N-(3-Dimethylaminopropyl)-N0-ethyl-Carbodiimide hydrochloride (EDC) succinic anhydride, Dimethylsulfoxide (DMSO), N-Hydroxy Succinimide (NHS), dimethyl amino pyridine (DMAP), N, N′-dicyclohexyl anhydrous 1,4-dioxane, and triethylamine (TEA) were obtained from Sigma Aldrich. All other chemicals were purchased from Emertat Chimi Company (Tehran, Iran).

### Synthesis of PEG-Arg@IONPs

**Figure 1:**
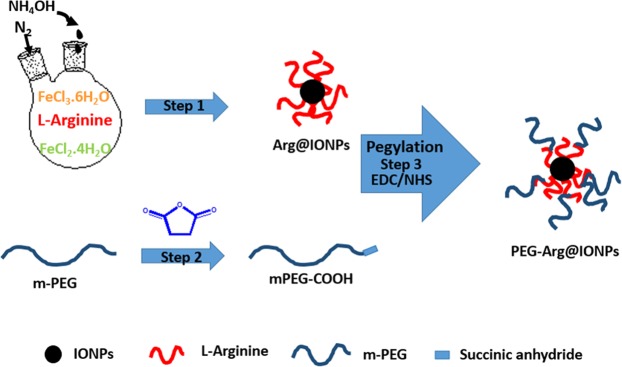
Schematic illustration of synthesis of PEG-Arg@IONPs.

The PEG-Arg@IONPs was synthesized in a three-step procedure. Schematic illustration of the synthesis of PEG-Arg@IONPs are shown in Fig. 1.

Step 1: Arg@IONPs were prepared based on a simple protocol. 150 ml of degased deionized H_2_O, 1.1 g of FeCl_3_⋅6H_2_O, 0.4 g of FeCl_2_⋅4H_2_O, and 2.79 g of L-Arginine were heated to 80 °C. 20 ml of ammonium hydroxide (NH_4_OH, 25%) was added dropwise to the mixture at N_2_ atmosphere under vigorous stirring on a magnetic stir plate. At the end of the reaction time, i.e. 6 h, the Arg@IONPs were separated by an external magnet under the beaker, and the resulting supernatant was discarded. The separated Arg@IONPs were washed several times with water using magnetic separation.

Step 2: The active carboxyl-terminus of mPEG (α-carboxy-*ω*-methoxy polyethylene glycol (mPEG-COOH)) was synthesized with succinic anhydride as described by Mathiyalagan *et al*.^25^. Briefly, mPEG-COOH was prepared as follows: 2 g of mPEG (MW 4000 g/mol; 0.5 mmol), 0.06 g of succinic anhydride (0.6 mmol), 0.061 g of DMAP (0.5 mmol), and 0.05 g of TEA (0.5 mmol) were mixed with 10 mL of anhydrous dioxane. The resultant solution was stirred at room temperature for 24 h. MPEG-COOH was precipitated with cold diethyl ether, then it was filtered, and at last, dried.

Step 3: Finally, the resultant mPEG-COOH conjugated to Arg@IONPs. mPEG-COOH (0.044 g, 0.02 mmol), EDC (0.06 g, 0.03 mmol), and DMAP were added to the stirred solution, in which DMAP was used as a catalyst and EDC and NHS as condensation agents. After 15 min, the Arg@IONPs were dispersed in deionized water, then added to the mixture, and stirred overnight. Following mPEG-COOH conjugation, the PEG-Arg@IONPs were separated magnetically, and the resulting supernatant was discarded. The separated PEG-Arg@IONPs were washed several times with water using magnetic separation, and then dried in a vacuum oven overnight.

### Characterization

The internal structure of PEG-Arg@IONPs was studied through transmission electron microscope (Cambridge 360–1990 Stereo Scan Instrument-EDS) measurements.

Morphology of PEG-Arg@IONPs was studied by atomic force microscopy (AFM) (JPK Nano wizard II, JPK instrument, Bouchestrasse, Berlin, Germany) in an intermittent contact mode.

X-ray diffraction was measured by a Bruker AXS model D8 Advance powder X-ray diffractometer.

FTIR spectra of the sample were measured by a Bruker, Tensor 27 FTIR spectrophotometer.

To evaluate the behavior in reaction to the increased temperature, thermogravimetric analysis (TGA, Linseis Instruments model, STA PT 1000, USA) and also, differential scanning calorimetry (DSC, Mettler Toledo, model Star SW 9.30, Schwerzenbach, Switzerland) were used.

Sample magnetization curves were attained by means of a vibrating sample magnetometer (VSM Magnetic Daghigh Daneshpajouh Co, Kashan, Iran).

ζ-potential and hydrodynamic size measurements were done using a nano/zetasizer (Malvern Instruments, Worcestershire, UK, model Nano ZS).

To quantify the stability of PEG-Arg@IONPs nanoparticles dispersed in water, DMEM, and saline, respectively, UV-visible absorbance at 450 nm were used in order to monitor the absorbance of the corresponding dispersion solution containing PEG-Arg@IONPs nanoparticles at a fixed wavelength similar to that in the reported study.

### *In vitro* study

#### Cell culture

Cell culture tests were done in two types of normal cell lines. The Human foreskin fibroblast (HFF2) cell line and Human embryonic kidney (HEK293) cell line were purchased from Pasteur Institute (Tehran, Iran). The cells cultured in 10% fetal bovine serum, 100 mg/ml of penicillin G and 100 mg/ml of streptomycin supplemented DMEM (Gibco, Germany) were kept in an incubator at 37 °C.

Cytotoxicity: The HEK 293 and HFF2 cells were used to study the cell toxicity. IONPs and PEG-Arg@IONPs in a dilution series were dispersed in DMEM (0.06, 0.09, 0.15, 0.19, 0.30 and 0.40 mg/mL NPs) and then, added to the wells of 96-well plate for 72 h. Control wells were treated with a free culture medium. At the end of the treatment, 20 µL of 3 MTT with 2.5 mg/mL concentration was added to the wells. Then, incubation continued for 4 h more, followed by detachment of MTT solution. Subsequently, 100 μL of DMSO was added to each well and next a microplate reader (1420 multilabel counter, Perkin Elmer, MA, USA) was used to read the absorbance at 570 nm.

#### Hemocompatibility

To examine the hemocompatibility, hemolysis assay of the human red blood cells (HRBCs) was performed. In fact, 1 mL of PEG-Arg@IONPs (suspended in PBS) at concentration of 10 mg/mL was added to 1 mL of the HRBCs (in PBS) solution. 1 mL of deionized water and 1 mL of PBS were used as positive and negative controls, respectively. The mixed solutions were put at 37 °C for 4 h, gently shaken. After centrifugation of the samples for 15 min at 13000 rpm, the absorbance of the supernatant was read at 545 nm by a microplate reader. Hemolysis percentage was calculated using the following relationship:

$$ \% \,{\bf{Hemolysis}}=\frac{{{\bf{A}}}_{{\boldsymbol{Sample}}}-{{\bf{A}}}_{{\boldsymbol{Negative}}}}{{{\bf{A}}}_{{\boldsymbol{Positive}}}-{{\bf{A}}}_{{\boldsymbol{Negative}}}}\times 100$$

Methods of this study were approved by Ethics Committee of Zanjan University of Medical Sciences, and participants signed an informed consent.

### *In vivo* study

All the methods used *in vivo* study were performed in accordance with relevant guidelines and regulations of Ethics Committee of Zanjan University of Medical Sciences under IR.ZUMS.REC.1396.307 ethical code.

#### MR Imaging

To conduct MRI scan in *ex vivo*, PEG-Arg@IONPs were injected to BALB/c mice (20 g) that were divided into 9 groups (n = 4): control, PEG-Arg@IONPs treated in 8 time intervals: 15 min, 30 min, 1 h, 2 h, 4 h, 6 h, 8 h, and 24 h after injection. PEG-Arg@IONPs were suspended in normal saline and then injected intravenously at a dose of 20 mg Fe/kg. At appropriate time intervals, the mice were anesthetized with ketamine. Then, they were sacrificed at programmed time intervals. Subsequently, the target organs, i.e. heart, liver, spleen and kidneys were extracted. Then the removed organs were briefly washed with saline and immersed in 4% paraformaldehyde. The removed organs were kept at 4 °C overnight. 1% Agarose gel was used to fix the collected organs in a plate prior to MR imaging. Images were taken by a Clinical 1.5 T whole body magnetic resonance scanner (Siemens Avanto Medical Systems, Berlin, Germany). TR/TE = 6440 ms/91 ms, FA = 90°, FOV = 2 cm × 2 cm, and slice thickness =1 mm are the imaging parameters.

Macro Imaging Software (version 1.47 v) was used for the analysis of the obtained images. Signal intensity of the extracted organs were obtained at the region of interest (ROI) of the organs image in the same slice on T_2_-weighted images before or after administration of PEG-Arg@IONPs at the programmed time intervals. The signal intensity of organs before (**SI*pre***) and after (**SI*post***) administration at programmed time intervals were applied to calculated the signal change percentage.

$$ \% \,{\bf{Signal}}\,{\bf{Change}}=\frac{{\bf{S}}{{\bf{I}}}_{{\boldsymbol{Pre}}}-{\bf{S}}{{\bf{I}}}_{{\boldsymbol{Post}}}}{{\bf{S}}{{\bf{I}}}_{{\boldsymbol{Pre}}}}\times 100$$

#### Stereological study

For hystomorphometric and hystopathological analysis, PEG-Arg@IONPs were injected into BALB/c mice (20 g) which were divided into 2 groups: control, treated with PEG-Arg@IONPs 15 day after injection. PEG-Arg@IONPs were suspended in normal saline and then injected intravenously at a dose of 20 mg Fe/kg. At proper time intervals, the mice were anesthetized with ketamine. Then, they were sacrificed at the programmed time intervals. Subsequently, the goal organs, i.e heart, liver, spleen and kidneys were extracted. The removed tissues were briefly washed with saline and then immersed in 4% formalin at least for 24 h. The fixed organs were embedded in paraffin, then sectioned and stained with hematoxylin and eosin (H&E).

Cell density estimation: Numerical density (Nv) of hepatocytes were calculated as follow:

$${\boldsymbol{Nv}}=\frac{\sum {\boldsymbol{Q}}}{\sum {\boldsymbol{P}}\times {\boldsymbol{h}}\times {\boldsymbol{a}}/{\boldsymbol{f}}}\times \frac{{\boldsymbol{t}}}{{\boldsymbol{BA}}}$$

where “ΣQ-” is the number of the nuclei, “h” is the height of disector, “a/f” is the frame area, “ΣP” is the total number of unbiased counting frame in all fields, “t” is the real section thickness measured in every field using microcator, and BA is the block advance of microtome, set at 10 μm^26^.

Volume estimation: The volumes of kidney (proximal and distal convoluted tubules), liver (sinusoid), spleen (whith pulp, red pulp), and heart (muscle fibers) were premeditated using Cavalieri method. Besides, their images were taken using stereological software designed at Stereology lab (Shahid Beheshti University of Medical Sciences), a grid of points was superimposed on the images. the volume of the tissue was assessed as follow:

$$V={\sum }^{}P\times \frac{a}{p}\times t$$

Where the “Σ*P*” was the total points hitting the tissue sections, “*a/p*” was the area associated with each point and “*t*” was the distance between the sampled sections^27^.

### Statistical analysis

Results were presented as mean ± SD. Statistical significance was determined using the one-way ANOVA tests, followed by the post-hoc Tukey. Statistical significance was set at p < 0.05.

### Ethical considerations

This study was approved by the Ethics Committee of the Zanjan University of Medical Sciences with IR.ZUMS.REC.1396.307 ethical code, and the study participants signed an informed consent. All methods were carried out in accordance with Ethics Committee of the Zanjan University of Medical Sciences guidelines and regulations. All experimental protocols were approved by Zanjan University of Medical Sciences and licensing committee.

## Results and Discussion

### Synthesis of PEG-Arg@IONPs

The Arg@IONPs, according to the literature, were synthesized by controlled simple co-precipitation procedure. Then, Arg@IONPs were conjugated with mPEG-COOH by ester covalent bond. MPEG-COOH was reactivated to its carboxylic end using EDC and NHS, and then conjugated with amine groups of Arg@IONPs. Active carboxyl-terminus of mPEG-COOH was synthesized by succinic anhydride.

### Characterization of PEG-Arg@IONPs

**Figure 2:**
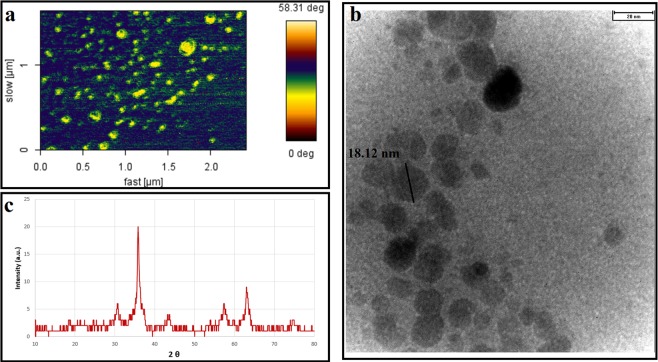
(**a**) AFM image of PEG-Arg@IONPs, (**b**) TEM image of PEG-Arg@IONPs, and (**c**) X-ray diffraction (XRD) pattern of PEG-Arg@IONPs.

**Figure 3:**
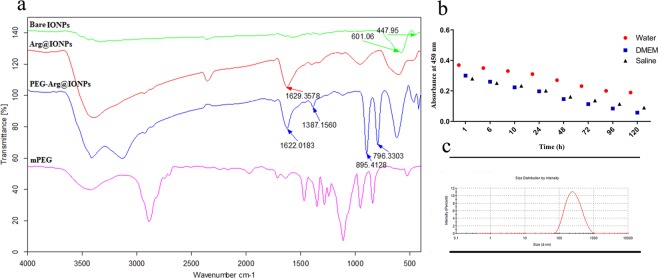
(**a**) FTIR spectra with some detailed bond peaks for bare IONPs, Arg@IONPs, PEG-Arg@IONPs, and mPEG. (**b**) UV-visible absorbance at 450 nm to screen dispersion stability of solutions containing PEG-Arg@IONPs in water, DMEM, and saline (**c**) Hydrodynamic size of PEG-Arg@IONPs.

The internal diameter of the formulated IONPs measured by TEM revealed the particle size of 18.02 ± 2.80 nm (mean ± SD (N = 18)) (Fig. 2b). The particle size measured by AFM was around 68.94 ± 8.70 nm (mean ± SD (N = 18)) (Fig. 2a). Hydrodynamic size measured by DLS was also around 230 nm (Fig. 3c)^28–32^.

Characteristic XRD peak for IONPs (Fig. 2c) was found at 2θ = 30.74°, 35.86°, 43.38°, 54.04°, 57.3°, and 63.02°, fitting to (220), (311), (400), (422), (422), and (511) Bragg reflection of Fe_3_O_4_ standards from a JCPDS file (PDF No. JCPDS 019-0629). IONPs had a cubic spinel structure not changeable during the production procedure.

Results of FTIR Confirmed that Arg and mPEG-COOH successfully conjugated to the surface of IONPs (Fig. 3a).

In FTIR spectra of Arg@IONPs, the C=O and C—O stretching vibrations were observed at ~1629 and ~1379 cm^−1^, respectively. Furthermore, overlaps of N—H stretching vibration with OH stretching can be seen at ~3400 cm^−1^. Moreover, in the spectra of PEG-Arg@IONPs, the absorption bands looking at 800–900 cm^−1^ were attributed to stretching vibration of C–O–C of PEG chains. Additionally, characteristic Fe–O bands (i.e. 450 and 610 cm^−1^) remained as it was in the PEG-Arg@IONPs.

**Figure 4:**
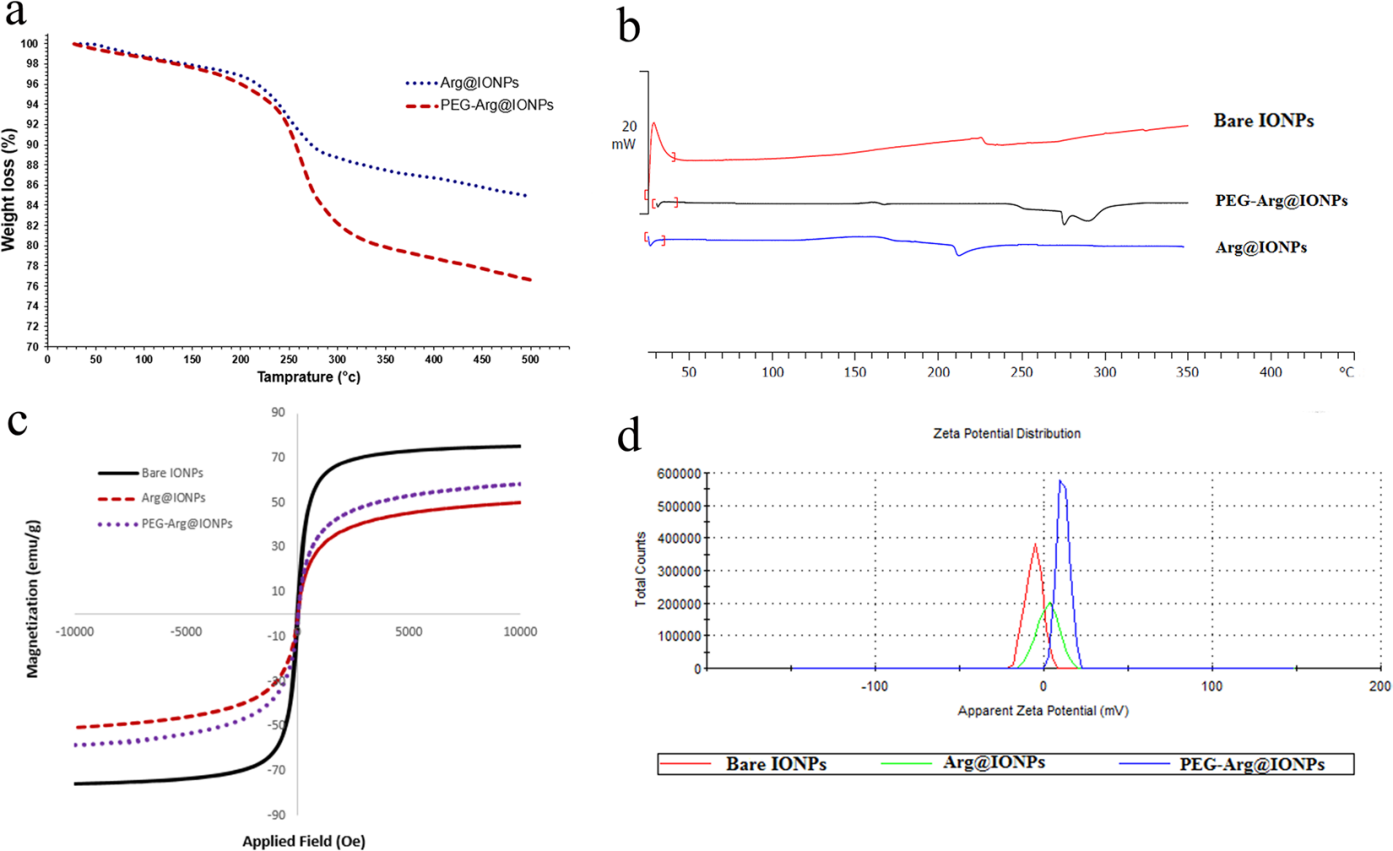
(**a**) TGA curves show the percentage of total weight loss of Arg@IONPs, and PEG-Arg@IONPs, (**b**) DSC thermograms of Bare IONPs, Arg@IONPs, and PEG-Arg@IONPs, (**c**) VSM curves of Bare IONPs, Arg@IONPs, and PEG-Arg@IONPs, (**d**) ζ-potential of Bare IONPs, Arg@IONPs, and PEG-Arg@IONPs.

Figure 4a shows TGA curves of Arg@IONPs and PEG-Arg@IONPs. For Arg@IONPs, net IONPs content was 84%, and the remainder was from Arg. For the PEG-Arg@IONPs, e net IONPs content was 76%. The remaining 24% was from Arg and PEG.

DSC can also offer supportive confirmation for the synthesis of PEG-Arg@IONPs. Figure 4b showed thermograms of bare IONPs, Arg@IONPs and PEG-Arg@IONPs.

Surface coating of IONPs can be approved by DSC. After coating of IONPs, melting points of Arg in Fig. 4 shifted to a lower temperature. In Arg@IONPs thermograms, an endothermic peak appeared at 153.22 °C, whereas the DSC thermogram of PEG-Arg@IONPs had an endothermic peak at ~ 280 °C. This single peak in PEG-Arg@IONPs thermogram confirmed the conjugation of PEG on the surface of Arg@IONPs.

Magnetic properties of IONPs, Arg@IONPs and PEG-Arg@IONPs were shown in Fig. 4c. Saturation magnetization (Ms) of Arg@IONPs and PEG-Arg@IONPs were found to be 58 emu/g and 49 emu/g, respectively. Ms of the modified IONPs were smaller than 75 emu/g of bare IONPs.

Change of surface charge of IONPs after modification and change in pegylation from −5.6 mV to 11.5 mV can be used as a further proof for coating the nanoparticles (Fig. 4d).

The stability of PEG-Arg@IONPs in water, DMEM, and saline were shown in Fig. 3b. UV-visible absorbance at 450 nm was used to screen dispersion stability of solution containing PEG-Arg@IONPs. Results obtained after five days showed that PEG-Arg@IONPs nanoparticles dispersed in water, DMEM, and saline lead to a slight decrease in UV-Visible absorbance, approving the excellent stability of these particles.

### Cytotoxicity assay

**Figure 5:**
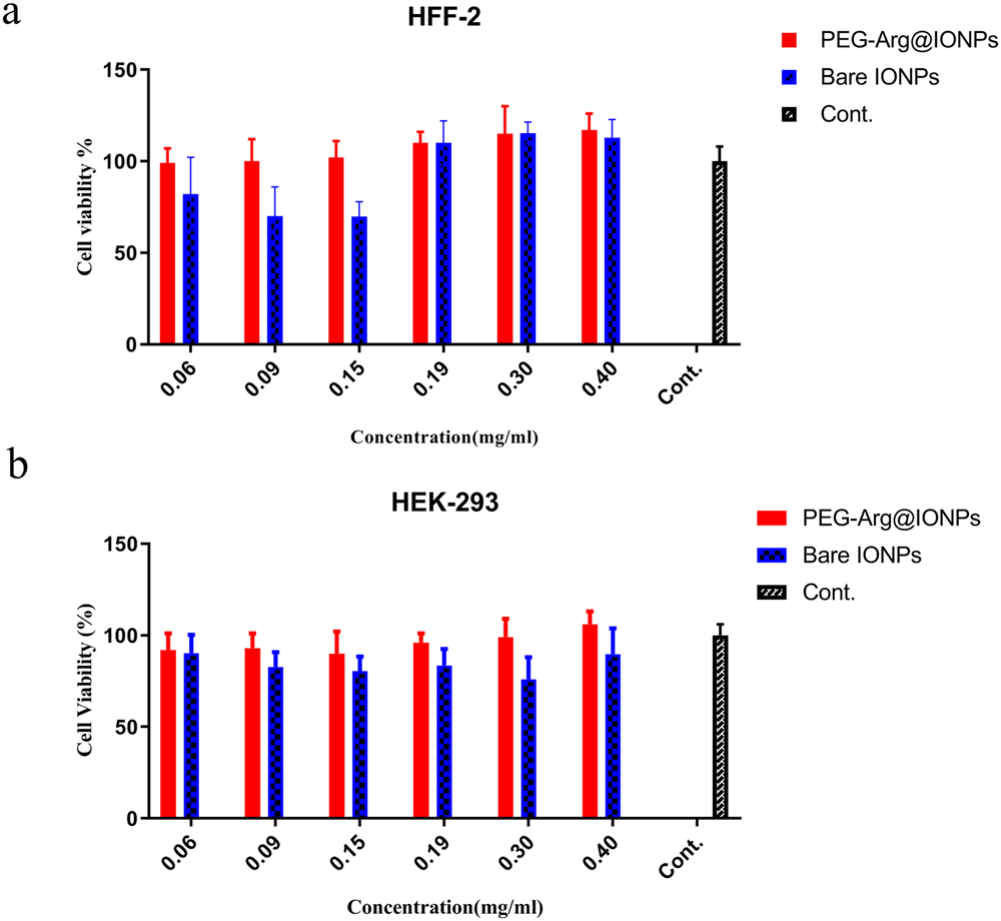
Cytotoxicity and metabolic activity analysis of Bare IONPs and PEG-Arg@IONPs after incubation at 72 h on (**a**) HFF-2, and (**b**) HEK-293 cell lines. Each bar represents the mean of five measurements ± SD.

The toxicity of PEG-Arg@IONPs has been one of the most essential issue for *in vivo* applications. Comparative cytotoxicity activity of IONPs and PEG-Arg@IONPs were done on HFF2 and HEK293 cells by a standard MTT colorimetric assay at different sample concentrations. Cell toxicity results were revealed in Fig. 5a,b. Cell lines were incubated with IONPs and PEG-Arg@IONPs for 72 h. MTT assays showed no noticeable inhibitive effects of PEG-Arg@IONPs against cell lines growth. The results showed that cell growth was not affected by PEG-Arg@IONPs.

Furthermore, blood adaptability of PEG-Arg@IONPs was obtained by *in vitro* hemolysis assay.

The PEG-Arg@IONPs did not affect HRBCs of the blood. The hemolytic activity was discovered to be less than 2.8%.

### *In vivo* Liver MRI study

**Figure 6:**
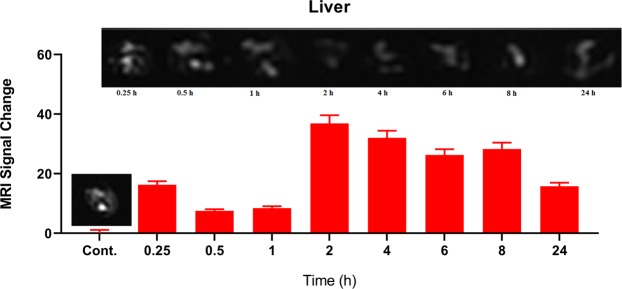
*In vivo* MRI studies: MRI signal trend and signal change intensity in liver at different time interval. Each bar represents the mean of five measurements ± SD.

In the previous works, it was shown that bigger IONPs administrated were picked up quickly by liver and spleen^33^. To study whether the PEG-Arg@IONPs could evade from reticuloendothelial system after administration of PEG-Arg@IONPs, the mice livers were removed and scanned by MRI at different programed time intervals. As seen in Fig. 6, one hour after injection, MRI image of liver section did not show any significant PEG-Arg@IONPs gathering (t signal change intensity of this organ 1 h after injection in Fig. 6 is less than 10); whereas, the signal change intensity of spleen, heart and kidneys confirmed that PEG-Arg@IONPs existed in other organs. In contrast, larger commercial IONPs, e.g. Resovist, were accumulated in the liver after administration^33^. PEG-Arg@IONPs had a long circulation time. The PEG-Arg@IONPs were found to be collected in liver after two hours *in vivo*. After 2 h, the liver was found to be significantly darker (Fig. 6) than that seen in the native scan.

After 2 h, the signal change intensity was found to be significantly larger than that of the control liver. We can realize this issue by significant darkening of liver 2 h after injection in comparison with the control.

### MRI monitored biodistribution and biodegradation

Using MRI monitoring, we can detect the presence of PEG-Arg@IONPs in kidneys, spleen, liver and heart (Figs 6 and 9). MRI measurements were done for 0–24 h. As the signal change intensity was sensitive to the local IONPs concentration, pre to post contrast administration, the increase in the signal change intensity indicated deposition of PEG-Arg@IONPs in the tissue.

**Figure 7:**
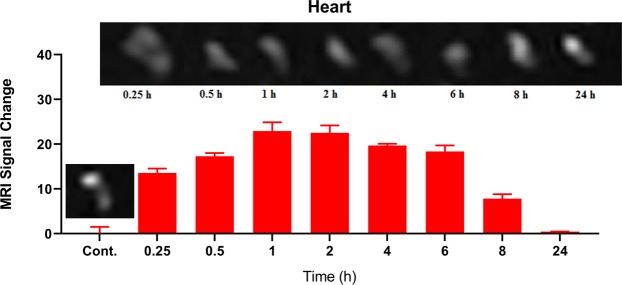
*In vivo* MRI studies: MRI signal trend and signal change intensity in heart at different time interval. Each bar represents the mean of five measurements ± SD.

**Figure 8:**
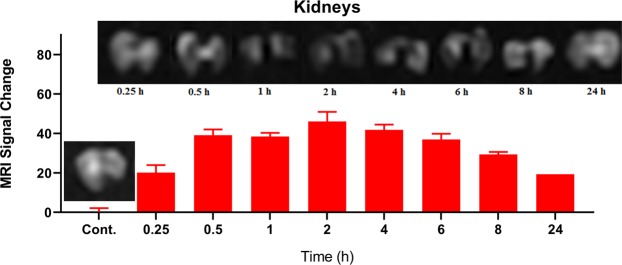
*In vivo* MRI studies: MRI signal trend and signal change intensity in kidneys at different time interval. Each bar represents the mean of five measurements ± SD.

**Figure 9:**
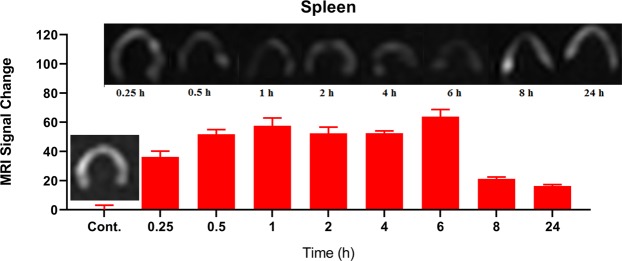
*In vivo* MRI studies: MRI signal trend and signal change intensity in spleen at different time interval. Each bar represents the mean of five measurements ± SD.

Figure 6 shows MR images of liver after injection of PEG-Arg@IONPs. 15 min, 30 min and 1 h after injection, the signal change intensity were less than the intensity obtained 2 h after the injection. However, the signal change intensity of other organs (e.g., the kidneys and heart) confirmed that PEG-Arg@IONPs existed in blood circulation.

After 2 hours, the signal change intensity was found to be significantly larger than that of the control liver. After 2 h, a strong and persistent darkening could be observed until the 8th hour, corresponding to the maximum contrast signal, while, the signal change intensity decreased after 24 h. Moreover, after 24 h, the signal change intensity was lower than that seen after 8 hours.

Figure 7 shows MR image of heart after injection of PEG-Arg@IONPs.

The Result showed that, as long as PEG-Arg@IONPs existed in blood circulation, darkening and signal change intensity of the heart was also more. For example, the signal change intensity value 1 hour after injection was 22.92; whereas, after 24 hours the signal change intensity in heart was approximately 0.44, suggesting that PEG-Arg@IONPs were banished from blood circulation. The results showed that nearly all IONPs were removed from blood circulation after 24 h.

Kidneys MR imaging and signal change intensity results showed that clearance by kidneys started from the injection moment (Fig. 8). Results confirm that kidneys are key organs responsible for the removal of PEG-Arg@IONPs from blood circulation.

Figure 9 shows spleen MR imaging after injection of PEG-Arg@IONPs.

PEG-Arg@IONPs were found to be collected in spleen after 30 min *in vivo*. After 30 min, a tenacious and constant darkening could be observed 6^th^ hour later, while the signal change intensity decreased after 8 h. It is likely that IONPs can be degraded by spleen.

So, according to MRI results, it is hoped that these PEG-Arg@IONPs may be used as T_2_ contrast agents. Consequently, the decreased signal change intensity during 24 h signified the biodegradation of IONPs from the body.

### Stereological study

The numerical density of hepatocytes was determined using optical disector method. Volumes of kidney (proximal and distal convoluted tubules), liver (sinusoid), spleen (white pulp, red pulp), and heart (muscle fibers) were calculated using Cavalieri method.

**Figure 10:**
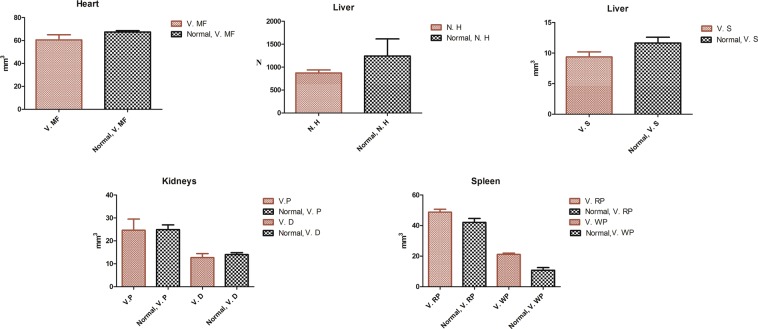
The numerical density of hepatocytes and volumes of kidney (proximal and distal convoluted tubules), liver (sinusoid), spleen (white pulp, red pulp), and heart (muscle fibers). Each bar represents the mean of four measurements ± SD. The data showed no significant difference between control and treated groups. V. MF: volume of muscle fiber in heart, N. H: number of hepatocytes in liver, V. S: Volume of sinusoid in liver, V. P and V. D: volume of proximal and distal tubules in kidneys, V. RP and V. WP: volume of red pulp and white pulp in spleen.

For stereological study of heart, the volume of muscle fiber was compared between the control group and the group treated with PEG-Arg@IONPs. The results showed that there was no significant difference between control and treated group as shown in Fig. 10. Moreover, PEG-Arg@IONPs did not show noticeable toxicity effect in heart muscle fiber.

For stereological study of liver, the number of hepatocytes and sinusoid volume were calculated by optical disector and cavalieri method, respectively. Stereological study results also showed no significant difference between control and treatment group, as shown in Fig. 10.

For stereological study of kidneys, proximal tubule and distal convoluted tubule volume of these organs were investigated in control group and the group treated with PEG-Arg@IONPs. Our study demonstrated that the difference between control and treatment group was not noticeable as shown in Fig. 10.

For stereological study of spleen, white pulp and red pulp volume in control group and the group treated with PEG-Arg@IONPs were also compared. Stereological study results showed no significant difference between them as shown in Fig. 10.

### Histopathological study

The liver, spleen, heart and kidney organs were taken from treated mice, 15 days after intravenous injection of PEG-Arg@IONPs, and after H & E staining was used for histological investigation.

**Figure 11:**
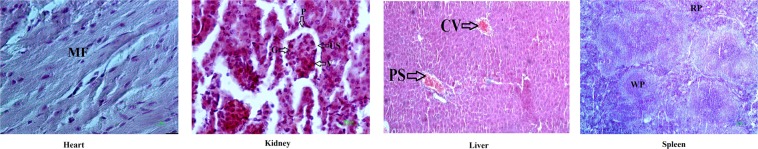
Histological study of the liver, spleen, heart, and kidneys after 15 days. MF: muscle fibers, P and V: Parietal and Visceral pleura, CV: Central vein, PS: Portal space, RP and WP: red pulp and white pulp.

As showed in Fig. 11, the harvested tissues were not affected by PEG-Arg@IONPs in comparison with the control animals. During 15 days of the trial, we did not detect any acute toxicity.

## Conclusion

In the current project, biocompatibility and biodegradation of IONPs were studied. PEG-Arg@IONPs were injected in mice and studied by MRI techniques. The results affirmed that there was no significant accumulation in liver at early hours and that nanoparticles had long circulation time. To the best of our knowledge, the stereological study of IONPs for investigation of biocompatibility and monitoring of biodegradation and distribution of IONPs were performed by MRI in this paper for the first time. Also, to validate biodegradation and clearance with body organs, qualitative evaluation of heart, kidneys, liver and spleen were done by MRI techniques at programed time intervals. Therefore, the studied PEG-Arg@IONPs can be one of the T_2_ contrast agents candidate for imaging the target organs.
